# Interaction Mechanism between Antibiotics and Humic Acid by UV-Vis Spectrometry

**DOI:** 10.3390/ijerph15091911

**Published:** 2018-09-03

**Authors:** Xiaoyu Yuan, Shengke Yang, Jie Fang, Xueli Wang, Haizhen Ma, Zongzhou Wang, Runze Wang, Yaqian Zhao

**Affiliations:** 1 Key Laboratory of Subsurface Hydrology and Ecological Effects in Arid Region, Ministry of Education, Chang’an University, Xi’an 710054, China; 15129037687@163.com (X.Y.); 18792975240@163.com (J.F.); wxl0120@126.com (X.W.); mhz9270@163.com (H.M.); zongzhouwang@126.com (Z.W.); nighyruo@126.com (R.W.); 2 Dooge Centre for Water Resource Research, School of Civil Engineering, University College Dublin, Belfield, Dublin 4 999014, Ireland; 2016229046@chd.edu.cn

**Keywords:** humic acid, oxytetracycline, sulfadiazine, UV-Vis spectrometry

## Abstract

In this study, the interaction between the humus and two antibiotics was studied by UV-Vis spectroscopy to describe the interaction mechanism and the effects of different environmental factors on the mechanism. Results showed that humic acid (HA) containing more aromatic groups was easily associated with antibiotics. In the HA-OTC, with the increase of the concentration of OTC, there were obvious absorption peaks in the 230–260 nm and 330–360 nm range, and the absorption band of the HA ultraviolet spectrum underwent a slight blue shift and the absorption intensity increased, demonstrating that a new ground state complex was generated. In the HA-SD, with the increase of SD concentration, an aromatic structure absorption peak appeared in the 190–220 nm range, and the peak value increased and the absorption band underwent a red shift, and the aromatization of HA decreased, which enhanced the interaction between the antibiotics and HA. With the increase of pH, the absorption band of HA, HA-OTC and HA-SD ultraviolet spectrum suffered a blue shift, the degree of polymerization of HA molecules decreased, and the number of adsorption binding sites increased, which resulted in the interaction of HA with antibiotics being enhanced. The absorption band of HA, HA-OTC and HA-SD displayed a red shift with the increase of ionic strength, which indicated that the repulsion within HA particles was weakened, and the molecular polymerization was strengthened and therefore, the interaction between antibiotics and HA was inhibited. The UV characteristics of the HA, HA-OTC and HA-SD systems were insensitive to the temperature. This study lays the foundation for better studying the effect of humus on the distribution of antibiotic residues in the environment.

## 1. Introduction

Antibiotics are widely used in medicine, animal husbandry, aquaculture, and personal care [1,2,3,4]. They cannot be completely metabolized in living organisms, and 25% to 75% of them are excreted into the environment in the form of the parent compound or its metabolites [5]. As an important kind of water pollutant, they are present in rivers [6,7], groundwater [8], and sediments [9] in many countries and pose a serious threat to the ecological environment and human health because of their resistance to degradation, water solubility and impacts on aquatic organisms. Among the many kinds of antibiotics, the largest residual amount corresponds to the tetracycline antibiotics (maximum content of 50 mg/kg) and the least residue corresponds to the sulfa antibiotics (maximum content of 1 mg/kg), however, sulfonamides have a stronger effect on the growth of plants [10,11]. Oxytetracycline (OTC) and sulfadiazine (SD) are the most typical representatives of tetracycline antibiotics and sulfa antibiotics, respectively. Because of their chemical stability, low solubility in water and difficulty of degradation in the environment, they can exist in water and sediments for a long time, which easily causes drug residues and pollution in the environment [12]. Therefore, they have gradually become a focus of organic pollution in the environment. A series of studies have shown that antibiotics will undergo a series of steps such as adsorption, migration, degradation and transformation [13,14,15,16] in the environment, largely influenced by the dissolved organic matter (DOM) and humus in the environment [17]. The reason is that DOM and humus have complex structures and many active groups, and can combine the antibiotics by hydrogen bonding, electrostatic interactions and cation exchange [18], thus contributing to the transformation of antibiotics in the environment [19,20].

The methods used to investigate the intercctions between humus and contaminants include potentiometric titration [21], voltammetry [22,23] and ion selective electrode methods [24,25]. Compared with these methods, the ultraviolet visible spectrum method is simple to operate, less destructive to the sample and less demanding, and the specific wavelength of the UV visible absorption ratio (E4/E6) is often used to indicate the structural changes of humus [26], which can provide information about humus molecules. At present, the use of the ultraviolet visible spectrum method to study the interaction between substances is mainly focused on the interaction of humic substances with heavy metals [27], while there is a lack of discussion with the mechanism of the interactions between humus and antibiotics as well as how they change with various environmental factors. Therefore, the use of the ultraviolet visible spectrum method to study the interaction mechanisms between humus and antibiotics could lay a foundation for the better understanding the effect of humus on the distribution of antibiotic residues in the environment. In this study, oxytetracycline (OTC) and sulfadiazine (SD) were used as the target pollutants and humic acid (HA) was selected as the representative of humus. The interaction between humic acid and two antibiotics as well as the effects of temperature, pH and ionic strength was studied by the ultraviolet visible spectrum method to explore their interaction mechanisms.

## 2. Materials and Methods

### 2.1. Instruments and Chemicals

Ultraviolet visible spectrophotometer (UV-2450, SHIMADZU, Jinan, China); total organic carbon analyzer (Vario EL cube, Elementar, Hanau, Germany); constant temperature water bath oscillator (SHA-C, Jingda, Changzhou, China).

The standard samples of oxytetracycline (OTC) and sulfadiazine (SD) were bought from the BBI Company (Vancouver, BC, Canada), the purity was USP grade (>95%); the experimental water was ultra-pure water; the other reagents were all analytically pure.

### 2.2. Preparation of Humic Acid

A certain amount of humic acid was dissolved with 2% NaOH solution and then filtered with a microporous filter membrane (0.45 μm) three times. The filtrate pH was adjusted to less than 1.5. The supernatant was removed after 30 min 2500 r/min centrifugation, cleaned with distilled water, and then centrifuged 10 min to remove the supernatant, dried and stored in the refrigerator (BCD-345WDGFU1, Haier, Qingdao, China). A certain amount of humic acid in a beaker is dissolved in 2% NaOH solution, the pH value was adjusted to about 8, and then filtered through a 0.22 μm microporous filter membrane, that is, the humic acid suspension was used as the stored liquid.

### 2.3. Experimental Methods

#### 2.3.1. UV-Vis Spectroscopy Experiments of Antibiotic Interactions with Humic Acid

With the addition of humic acid and different volume of antibiotics in a 10 mL slug colorimetric tube, the concentration of humic acid was 20 mg/L and the concentration of the antibiotic was 1.5–9.0 mg/L. After 30 min in a 25 °C water bath, the UV visible absorption spectrum of the mixture of humic acid and various concentrations of antibiotics was determined. Under the same conditions, the concentration of antibiotics was 6.0 mg/L, while the concentration of humic acid was changed to 5–30 mg/L.

#### 2.3.2. Fluorescence Quenching Experiments of Humic Acid and Antibiotics

With the addition of humic acid and different volume of antibiotics in the 10 mL slug colorimetric tube, the concentration of humic acid was 20 mg/L and the concentration of the antibiotics was 0, 3.0, 6.0, 9.0 mg/L. After 30 min in a 25 °C water bath, the three-dimensional fluorescence spectrum of the humic acid was determined.

#### 2.3.3. Environmental Factors Impact on the Experiment

The humic acid and different volumes of antibiotics were added to a 10 mL slug colorimetric tube, and the deionized water was added, so the concentration of humic acid was 20 mg/L, and the concentration of antibiotics was 6.0 mg/L. The experiments were carried out in a constant temperature water bath at 15, 25 and 40 °C for 30 min. Under the same conditions, the pH was adjusted to 4–10 and the solutions held for 30 min at 25 °C to display the pH impact. The ionic strength gradient of 0.1 mol/L NaCl solution system was 0.0025–0.08 mol/L, and the other steps were the same as in the pH effect experiments.

## 3. Results and Discussion

### 3.1. UV-Visible Spectroscopy Analysis of Antibiotics and HA

**Figure 1:**
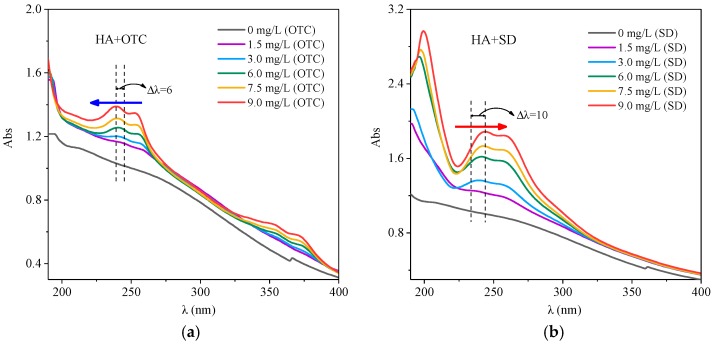
The interactions of different concentrations of OTC and SD with HA (**a**) OTC; (**b**) SD.

The UV Vis spectra after adding different concentrations of OTC and SD solution to HA solution, are shown in Figure 1. From Figure 1a, it can be seen that after adding OTC, obvious absorption peaks appeared in the 230–260 nm and 330–360 nm range, and with the OTC concentration increased, the two peaks had an obvious color enhancement effect, while the peak position showed a weak blue shift. This indicated that the interaction between HA and OTC produced a new ground state complex, thus changing the UV Vis spectra of the system.

As shown in Figure 1b, after the addition of SD, the spectrogram appeared to have an obvious absorption peak in the 190–220 nm range, which may due to the interaction of SD and HA, leading to an absorption peak near 210 nm resulting from an aromatic structure, and with the increase of SD concentration, the peak value became larger and the peak a bit red shifted. This may result from the reduction of the degree of aromatization of HA with the addition of SD, leading to an increase in the exposed chromophores and color-assisting groups of HA [28], which combine with SD to change the UV visible spectrum of the system.

**Figure 2:**
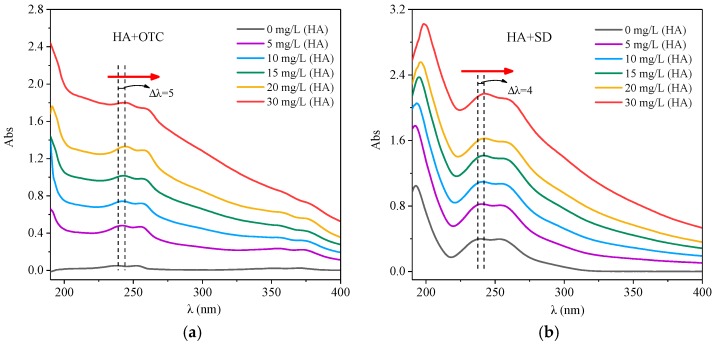
The interactions of different concentrations of HA with OTC and SD. (**a**) OTC; (**b**) SD.

After fixing the concentration of OTC solution and adding different concentrations of HA solution, the UV visible spectrogram of the system is shown in Figure 2. In Figure 2a, the absorbance of the HA-OTC system increased evenly with the increase of the absorption wavelength, and there was a stronger absorption peak in the 230–260 nm range, and also a weaker peak in the 330–360 nm range. With the increase of HA concentration, the absorbance increased gradually and the peak position was weakly red shifted. This may be due to the increase of the mutual agglomeration of HA molecules with the increase of concentration, thus changing the spectral characteristics. The red position shift may be due to the steric hindrance in the molecular system, resulting in an increase of the degree of conjugation [29].

It was obtained from Figure 2b that after adding different concentrations of HA solution, the HA-SD system had no new absorption peaks compared with the single SD system, but with the increase of HA concentration, the absorbence increased gradually and the peak position showed a little red shift. One probable reason was that the π bond interaction between the intramolecular phase generated a large π bond, the electron delocalization to a number of atoms led to the reduction of the energy of the π–π* because the distance between the energy levels of the large π bond was close [30]. The other reason was that the SD was bound to the reactive functional groups of HA, which can promote the polymerization of HA colloidal particles and reduce the spacing of the chromophore group benzene ring, resulting in a red shift of the peak position [27]. This indicated that the concentration of HA affects the internal structure of HA molecules, thus affecting the strength of the SD interactions.

### 3.2. Fluorescence Evidence of Interactions between the Different Antibiotics and HA

**Figure 3:**
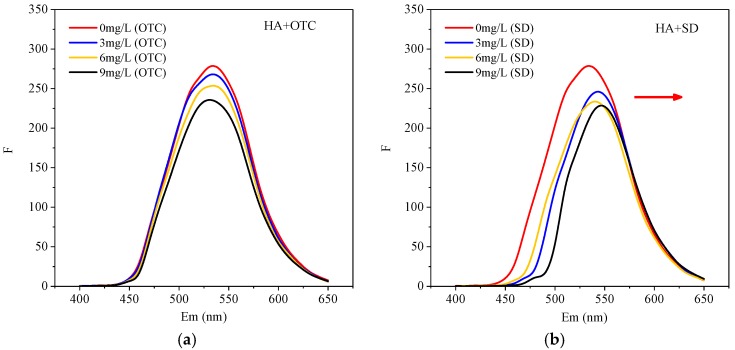
The fluorescence intensity (Ex/Em = 375nm/400–650nm) of HA with the presence of different concentration of (**a**) OTC and (**b**) SD.

In order to verify the interaction between HA and antibiotics, different concentrations of OTC and SD were added to the HA solution, and the fluorescence spectra of the system were determined, as shown in Figure 3.

It can be seen from Figure 3a that the addition of OTC had no significant effect on the three-dimensional fluorescence peak shape and peak position of the HA-OTC system compared with HA. However, as the concentration of OTC increased, the intensity of the fluorescence emission spectrum decreased regularly. This indicated that the addition of OTC caused HA fluorescence quenching. Interactions had taken place between them, and the polarity around the HA fluorophore had also changed [31]. It can be seen from Figure 3b that after adding SD, the peak shape of the HA-SD system was basically unchanged. However, as the concentration of SD increased, the intensity of the fluorescence emission spectrum decreased, and the peak position shifted significantly, indicating a strong interaction between SD and HA. The fluorescence signal and concentration can be fitted by the Stern-Volmer model [32]:

F_0_/F = 1 + Kqζ0[Q] = 1 + Ksv[Q] (1)

**Figure 4:**
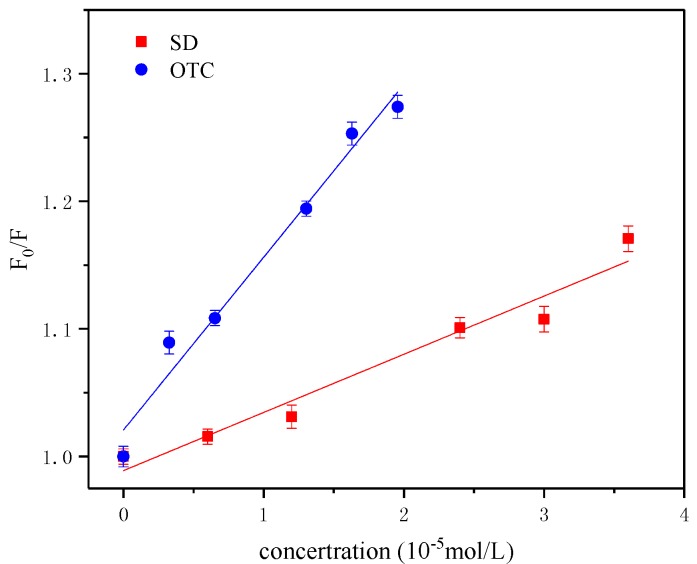
Stern-Volmer plots of OTC and SD for HA at temperature of 25 °C.

The results are shown in Figure 4. The change was a single line, indicating that both antibiotics displayed a single quenching process. The interaction constants of the different antibiotics with HA were obtained as shown in Table 1, where the quenching rate constants Kq of OTC and SD for HA are 9.811 × 10^11^ L·mol^−1^·s^−1^ and 5.271 × 10^11^ L·mol^−1^·s^−1^, respectively, which were larger than the quenching constant of the maximum diffusion collision (2.0 × 10^10^ L·mol^−1^·s^−1^), so the effect of OTC and SD on HA was a single static quenching of non-luminescent complexes. The fluorescence quenching constant Ksv and the binding constant Kb of OTC were greater than those of SD, which showed that OTC has a stronger quenching effect on HA, and the interaction was stronger. Moreover, the number of the binding points of OTC, SD and HA interactions were 1.207 and 0.997, respectively. It was shown that a conjugate was formed between HA-OTC and HA-SD in a 1:1 ratio. This type was similar to the interaction between HA and metal ions, which may be due to the fact that HA conformation and molecular rigidity have been changed during fluorescence quenching [33,34]. This conclusion and the movement of the absorption peaks (red shift, blue shift) in the UV spectrum analysis demonstrated that interactions between antibiotics and HA can occur.

### 3.3. The Effect of Temperature on the Action of Antibiotics and HA

**Figure 5:**
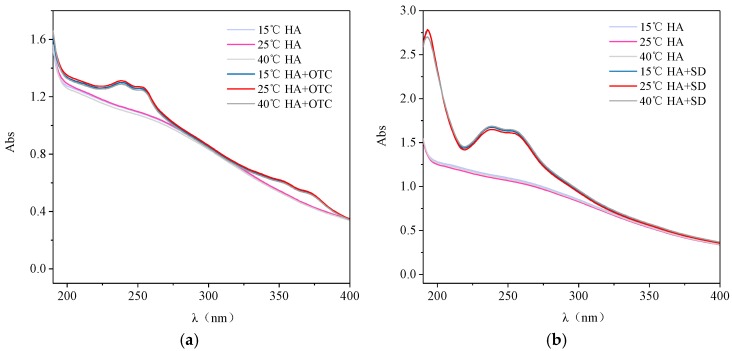
The interactions of OTC and SD with HA under different temperature. (**a**) OTC and (**b**) SD.

The temperature effects on the UV Vis spectra of HA-OTC and HA-SD systems are shown in Figure 5. Compared with the single HA system, the absorbance of the HA-OTC system was enhanced and the absorption peaks appeared in the range of 230–260 nm and 330–360 nm (Figure 5a), which may be due to the interaction of OTC and HA forming a new ground state complex, which affected the UV visible spectrum of the system. However, with the increase of temperature, both the HA-OTC system and the single HA system showed no changes in the absorbance intensity and peak position, indicating that the temperature had little effect on the HA and HA-OTC systems. 

As *seen in* Figure 5b, the absorbance of the HA-SD system was enhanced compared with the single HA system, and an absorption peak from the aromatic structure appeared near 210 nm, which indicated that the addition of SD reduces the degree of aromatization of HA molecules, exposing more chromophore and chromaticity groups [28]. Otherwise, with the change of temperature, there was no obvious change in the UV-visible spectrum intensity and peak position of the HA-SD system, indicating that the temperature has hardly any effect on the interaction of HA-SD.

### 3.4. Effect of PH on Antibiotic and HA Action

#### 3.4.1. Effect of PH on HA UV-Vis Spectra

**Figure 6:**
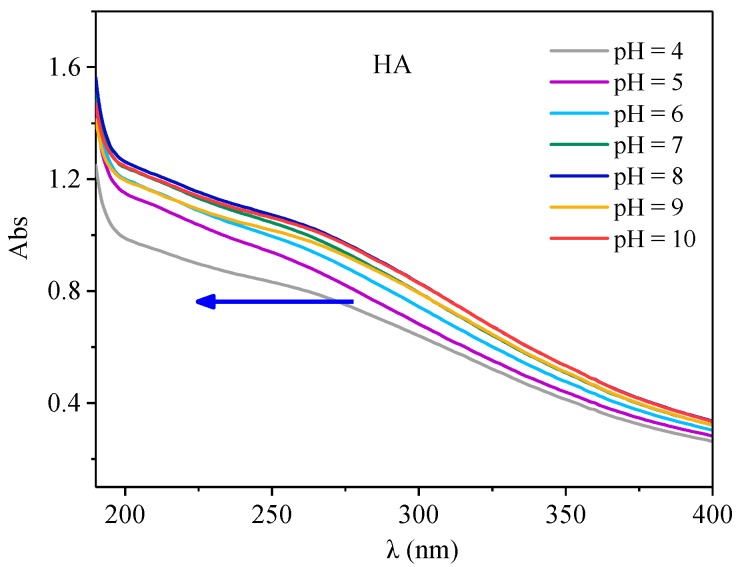
The UV-visible spectra of HA under different pH conditions.

The UV visible spectra of HA molecules under different pH conditions are shown in Figure 6. As a whole, with the increase of absorption wavelength, the absorption intensity decreases rapidly and had no obvious absorption peak, but there was a more obvious absorption platform within the 230–280 nm range, which may be the aromaticity step. Similar phenomena were also found by Guo [35]. In addition, with the increase of pH, the absorption strength of HA gradually increased, and a weak blue shift occurred, which was similar to the results of Yang [27]. This may be related to the changes of the acid groups in organic structures and their molecular configurations with the change of pH. According to the changes of UV spectra under different pH conditions, it is known that HA has aromatic structural groups, which can interact with antibiotics, and pH has a certain influence on its structure, so it may affect the interactions between HA and antibiotics.

#### 3.4.2. Effect of pH on the Interaction between Two Antibiotics and HA

**Figure 7:**
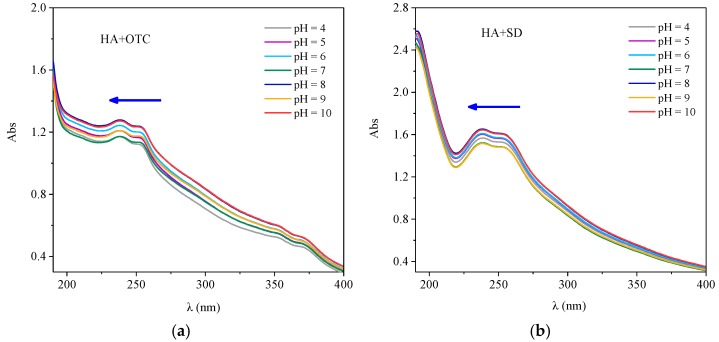
The interactions of OTC and SD with HA under different pH conditions. (**a**) OTC; (**b**) SD.

**Figure 8:**
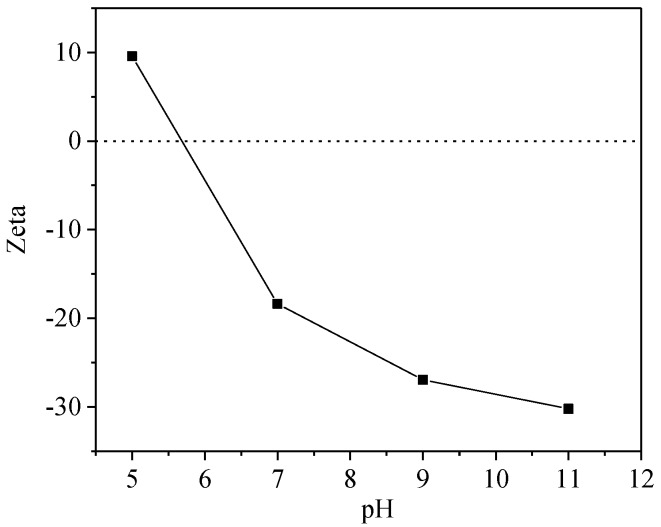
Zeta potential of HA-antibiotic systems under different pH conditions.

The UV Vis spectra of HA-OTC and HA-SD systems with different pH values are shown in Figure 7. As obtained from Figure 7a, the spectral peak type of HA-OTC at different pHs was basically unchanged, and there was a distinct and weaker acromion in the range of 230–260 nm and 350–370 nm. With the increase of pH, an obvious color enhancement effect and a slight blue shift of the peak position were seen in the spectrogram. In a further investigation of the interactions of HA and antibiotics in soil and water systems, the variation of the zeta potential at different pH values is shown in Figure 8. As the pH increased, the absolute value of the zeta potential of the HA and antibiotic interaction increased gradually. Mainly because of the increase of pH, the acid functional groups of HA were dissociated continuously, the absolute value of the zeta potential increased, the repulsive force between the HA molecules and the particles increases, the intramolecular and intermolecular hydrogen bonds of the HA colloid were broken, resulting in a decrease in the degree of polymerization of the HA particles [36], which leads to an increase of the energy needed for the electron transitions of HA molecules. As the degree of polymerization of HA decreased, the specific surface area increased, thus enhancing the adsorption and binding ability with OTC.

As obtained from Figure 7b, the peak type of the spectrum of the HA-SD system at different pH values was basically unchanged, and obvious absorption peaks appeared in the 190–210 nm and 230–260 nm range. With the increase of pH, the absorption intensity of the whole spectrum was gradually enhanced and a blue shift of the peak position occurred. It was possible that the carboxyl and hydroxyl groups in the HA molecules were mostly dissociated when the pH rises, producing a large number of negative charges, extending the configuration of HA molecules, whereby the p–π conjugation of the whole system was greatly enhanced, the electron transition probability increased, and the absorption strength increased and the peak position blue shift was affected [37]. This indicated that the interaction between HA and SD was enhanced under alkaline conditions.

#### 3.4.3. Effect of pH on E4/E6 Values

The UV visible absorption ratio of specific wavelengths is often used to indicate the structural changes of humus, and the one most widely used is E4/E6 [38,39,40], which can reflect the degree of condensation, aromatization, and molecular complexity of the C skeleton of the humus. It is known from Section 2.3.1 that the change of pH value has great influence on the UV visible spectrum of HA. Therefore, it was necessary to study the influence of different pH on the individual system of HA and the E4/E6 value of HA-OTC and HA-SD systems.

**Figure 9:**
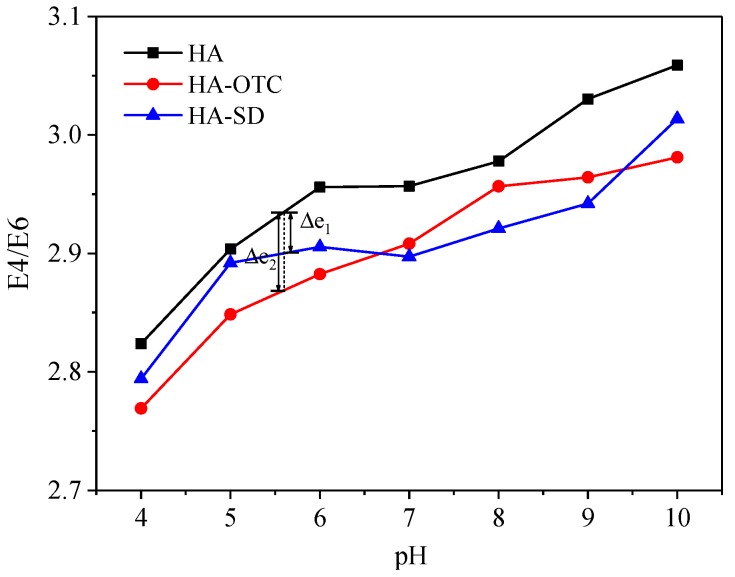
The E4/E6 of HA under different pH conditions.

The E4/E6 ratio pattern of HA, HA-OTC, and HA-SD systems under different pH is shown in Figure 9. It can be seen that with the increase of pH, the E4/E6 ratio increased gradually, indicating that with the increase of pH, the degree of skeleton polymerization or the degree of carbonyl conjugation of HA decreased, which was beneficial to the combination with antibiotics and HA [28]. Moreover, under the same pH conditions, the E4/E6 values of HA-OTC and HA-SD were less than HA, and under acidic conditions, the reduction amplitude (Δe_1_) of HA-SD was significantly smaller than that of HA-OTC (Δe_2_), which showed that the interaction between HA and SD was weaker than HA and OTC when the pH was between 4 and 6.8. It may be due to the fact that SD contains more amino groups and can bind to H+ under acidic conditions, thereby repelling the carboxyl groups on HA.

### 3.5. Effect of Ionic Strength on Antibiotics and HA

#### 3.5.1. Effect of Ionic Strength on UV-Visible Spectra of HA

**Figure 10:**
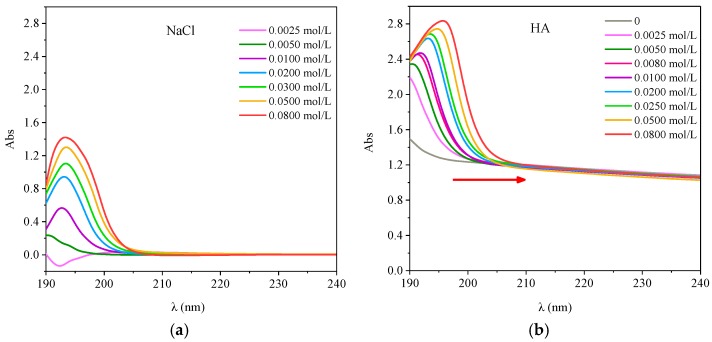
UV-visible spectra of the different concentration of NaCl and HA with different ionic strength. (**a**) OTC; (**b**) SD.

The UV Vis spectra of HA with different concentrations of NaCl is shown in Figure 10. The UV-visible spectrum of NaCl showed a characteristic absorption peak at about 193 nm. With the increase of NaCl concentration the absorbency increased gradually (Figure 10a). As shown in Figure 10b, when the ionic strength was 0, the absorption spectrum of HA decreased with increasing wavelength, and no obvious absorption peaks were observed. When ions existed, there was an obvious absorption peak in the range of 190–200 nm.

With the increase of ionic strength, the peak intensity increased and the peak position was red shifted. It was likely to the increase of the ion concentration in the solution leading to the repulsion between the HA molecules and the particles increased, strengthening the polymerization of the HA molecules, which led to the red shift of the absorption band and the increase of the absorption strength [27]. In addition, the chromogenic groups of the molecules were not conjugated by the effect of the stereoscopic effect, but their electron clouds interacted with each other because of their arrangement in space, which led to the change of the position and strength of the UV absorption bands [29]. This indicated that changes in ionic strength affect the structural changes of HA molecules and inhibited their interaction with antibiotics.

#### 3.5.2. Effect of Ionic Strength on the Interaction between the Two Antibiotics and HA

**Figure 11:**
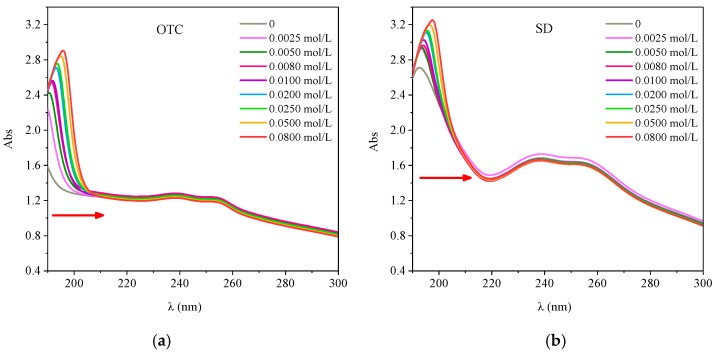
The interactions of OTC and SD with HA under different ionic strength. (**a**) OTC; (**b**) SD.

The UV Vis spectra of HA-OTC and HA-SD systems with different ionic strength are shown in Figure 11. According to Figure 11a, when the ionic strength was 0 mol/L, the absorbance of the HA-OTC system decreased slowly with the increase of wavelength, and a weak absorption peak appeared in the 230–260 nm range. With the increase of ionic strength, a clear absorption peak appeared in the 190–200 nm range and the absorbency increased continuously and the peak position was red shifted, which was similar to the study of Lan [41]. It was possible that the ions produced by the ionizing ions were competing with the complexation of OTC on HA when the ionic strength increased, thus producing a new peak for the multisubstituted phenolics and the π–π* transition of the benzene carboxylic group, the conjugation degree of the system was increased at the same time, and the molecular polymerization was strengthened. Although the groups were not conjugated, their electronic clouds interacted with each other due to their arrangement in space, resulting in the change of the location and intensity of the ultraviolet absorption band [29]. Therefore, the increase of ionic strength strengthened the polymerization of HA molecules and weakened the interaction with OTC.

As shown in Figure 11b, when the ionic strength was 0 mol/L, the spectral map of the HA-SD system had a distinct absorption peak at 192 nm, and a wider but less intense absorption peak appeared in the 230–260 nm range. With the increase of the ion intensity, the absorbency increased and the absorption peak of the 192 nm was red shifted. This may be due to the increase of ionic strength and the binding of the ionized cations with the active functional groups of HA, the decrease of the electron density on the HA molecules, the weakening of the repulsive force between the HA micelles and the molecules, and the strengthening of the polymerization of the HA colloid, thus reducing the distance of the phenyl ring of the chromogenic group [27]. The ionic strength increased and the ionized cations played a competitive role in the binding sites of HA and inhibited the interaction between HA and SD.

#### 3.5.3. Effect of Ionic Strength on E4/E6 Values

**Figure 12:**
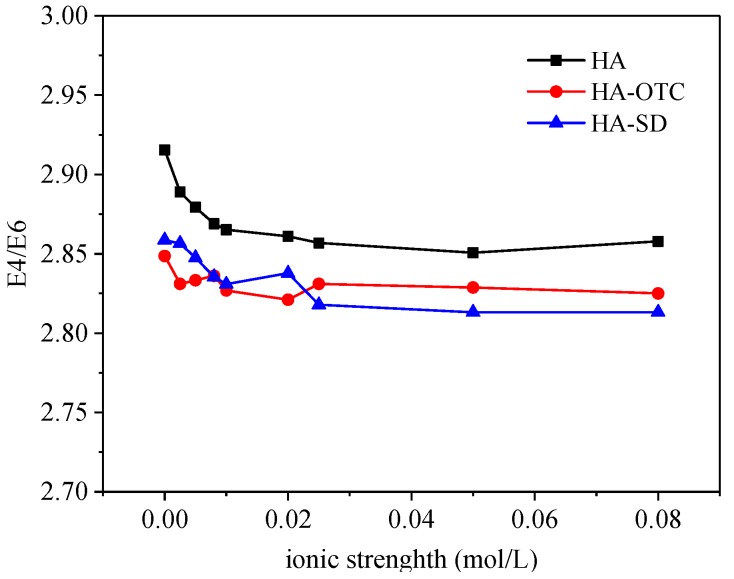
The E4/E6 of HA under different ionic strengths.

The E4/E6 ratio of HA, HA-OTC and HA-SD under different ionic strengths is shown in Figure 12, where when ionic strength was less than 0.02 mol/L, E4/E6 decreased slowly with the increase of ionic strength, indicating that the degree of skeleton polymerization or the conjugation degree of the carbonyl group increased, which inhibited the interaction between HA and antibiotics. When the ionic strength of the solution was 0.02 mol/L–0.08 mol/L, the E4/E6 ratio basically had no obvious change, which showed that the ion intensity change had little effect on the E4/E6 ratio in this range.

## 4. Conclusions

The current study revealed that HA could easily bind to antibiotics because of its aromatic groups. As the HA concentration increased, the reduction of the degree of polymerization of the C skeleton in HA benzene rings or the carbonyl groups was more favorable for its interaction with antibiotics. In the HA-OTC system, as the OTC concentration increased, the peak intensity increased and the position was weakly blue-shifted, because the interaction between HA and OTC produced a new ground state complex. However, as the SD concentration increased, the peak intensity increased and the position was weakly red-shifted, because the addition of SD decreased the degree of aromatization of HA, increasing the number of chromophores and helper groups exposed on the HA surface.

Environmental factor impact experiments showed that both of the pH and ionic strength had a significant effect on the interaction of HA with antibiotics whereas temperature has little effect. With the increase of pH, the absorbance of HA, HA-OTC and HA-SD systems increased, the peak position was blue-shifted and the E4/E6 values of HA-OTC and HA-SD systems increased gradually, suggesting that the degree of polymerization of HA molecules was reduced, so the configuration was extended and the specific surface area increased, which can provide more adsorption binding sites and the interaction with antibiotics was enhanced, while with the increase of ionic strength, the absorbance of HA, HA-OTC and HA increased, the peak position was red-shifted, and E4/E6 decreased weakly, which inhibited the interaction with antibiotics on the contrary.

## Figures and Tables

**Table 1:** The fitted association constant of the interaction between HA OTC and SD.

Quencher	Ksv (10^3^·L·mol^−1^)	Kq (10^11^·L·mol^−1^·s^−1^)	Kb (10^3^·L·mol^−1^)	*n*
OTC	9.811	9.811	7.183	1.207
SD	5.271	5.271	3.123	0.997

## References

[1] Islas-Espinoza M., Wexler M., Bond P.L. (2012). Soil Bacterial Consortia and Previous Exposure Enhance the Biodegradation of Sulfonamides from Pig Manure. Microb. Ecol..

[2] Juan-García A., Font G., Picó Y. (2010). Simultaneous determination of different classes of antibiotics in fish and livestock by CE-MS. Electrophoresis.

[3] Nicolaou K.C., Boddy C.N., Bräse S., Winssinger N. (1999). Chemistry, Biology, and Medicine of the Glycopeptide Antibiotics. Angew. Chem. Int. Ed..

[4] Wang Z., Zhang X.H., Huang Y., Wang H. (2015). Comprehensive evaluation of pharmaceuticals and personal care products (PPCPs) in typical highly urbanized regions across China. Environ. Pollut..

[5] Sarmah A.K., Meyer M.T., Boxall A.B. (2006). A global perspective on the use, sales, exposure pathways, occurrence, fate and effects of veterinary antibiotics (VAs) in the environment. Chemosphere.

[6] Hirsch R., Ternes T., Haberer K., Kratz K.L. (1999). Occurrence of antibiotics in the aquatic environment. Sci. Total Environ..

[7] Petrović M., Hernando M.D., Díazcruz M.S., Barceló D. (2005). Liquid chromatography-tandem mass spectrometry for the analysis of pharmaceutical residues in environmental samples: A review. J. Chromatogr. A.

[8] Sacher F., Lange F.T., Brauch H.J., Blankenhorn I. (2001). Pharmaceuticals in groundwaters analytical methods and results of a monitoring program in Baden-Wurttemberg, Germany. J. Chromatogr. A.

[9] Zhang Y., Xu J., Zhong Z., Guo C., Li L., He Y., Fan W., Chen Y. (2013). Degradation of sulfonamides antibiotics in lake water and sediment. Environ. Sci. Pollut. Res. Int..

[10] Zhao L., Dong Y.H., Wang H. (2010). Residues of veterinary antibiotics in manures from feedlot livestock in eight provinces of China. Sci. Total Environ..

[11] Wu N., Qiao M., Zhang B., Cheng W.-D., Zhu Y.-G. (2010). Abundance and Diversity of Tetracycline Resistance Genes in Soils Adjacent to Representative Swine Feedlots in China. Environ. Sci. Technol..

[12] Li Z.J., Qi R.H., Long J., Fan F.F., Fan F.L., Liang Y.C. (2010). Influence of particle size on the adsorption of oxytetracycline on black and red soils. J. Agro-Environ. Sci..

[13] Bing L., Tong Z. (2010). Biodegradation and adsorption of antibiotics in the activated sludge process. Environ. Sci. Technol..

[14] Chen W.R., Huang C.H. (2010). Adsorption and transformation of tetracycline antibiotics with aluminum oxide. Chemosphere.

[15] Wang R., Yang S., Fang J., Wang Z., Chen Y., Zhang D., Yang C. (2018). Characterizing the Interaction between Antibiotics and Humic Acid by Fluorescence Quenching Method. Int. J. Env. Res. Public Health.

[16] Zhang X., Xiang L., MO C.H., Li W.Y., Cai Q.Y., Huang X.P., Wu X.L., Li H. (2014). Migration Behavior and Influence Factors of Quinolone Antibiotics in Soil. J. Agro-Environ. Sci..

[17] Blackwell P.A., Kay P., Ashauer R., Boxall A.B.A. (2009). Effects of agricultural conditions on the leaching behaviour of veterinary antibiotics in soils. Chemosphere.

[18] Carmosini N., Lee L.S. (2009). Ciprofloxacin sorption by dissolved organic carbon from reference and bio-waste materials. Chemosphere.

[19] Ling W., Sun R., Gao X., Xu R., Li H. (2015). Low-molecular-weight organic acids enhance desorption of polycyclic aromatic hydrocarbons from soil. Eur. J. Soil Sci..

[20] Amir S., Jouraiphy A., Meddich A., El G.M., Winterton P., Hafidi M. (2010). Structural study of humic acids during composting of activated sludge-green waste: Elemental analysis, FTIR and 13C NMR. J. Hazard. Mater..

[21] Sikora F.J., Stevenson F.J. (1988). Silver complexation by humic substances: Conditional stability constants and nature of reactive sites. Geoderma.

[22] Carballeira J.L., Antelo J.M., Arce F. (2000). Analysis of the Cu^2+^-soil fulvic acid complexation by anodic stripping voltammetry using an electrostatic model. Environ. Sci. Technol..

[23] Ramos M.A., Fiol S., López R., Antelo J.M., Arce F. (2002). Analysis of the effect of pH on Cu^2+^-fulvic acid complexation using a simple electostatic model. Environ. Sci. Technol..

[24] Cao J., Lam K.C., Dawson R.W., Liu W.X., Tao S. (2004). The effect of pH, ion strength and reactant content on the complexation of Cu by various natural organic ligands from water and soil in Hong Kong. Chemosphere.

[25] Christl I., Milne C.J., Kinniburgh D.G., Kretzschmar R. (2001). Relating ion binding by fulvic and humic acids to chemical composition and molecular size. 2. Metal binding. Environ. Sci. Technol..

[26] Chen Y., Senesi N., Schnitzer M. (1977). Information Provided on Humic Substances by E4/E6 Ratios. Soil Sci. Soc. Am. J..

[27] Yang Y., Lan Y., Jin P., Wang X. (2015). Effects of pH, ionic strength and heavy metal ions on UV spectras of humic acid. Chinese J. Environ. Eng..

[28] Xu Y., Zhanf H., Kan L., Zhou Q., Luo M., Chen B. (2010). Investigation on the Interaction of Tetrabutyltin Compound and Humic Acids by Spectroscopy. GuangZhou Chem. Indus..

[29] Sato O., Kumada K. (1967). The chemical nature of the green fraction of P type humic acid. Soil Sci. Plant Nutr..

[30] Fu L., Liu X.F., Zhou Q.X., Zhang J.X., Dong J.Y., Wang J.F. (2014). Characterization of the interactions of human serum albumin (HSA), gatifloxacin, and metronidazole using spectroscopic and electrochemical methods. J. Lumin..

[31] Jiang A.W., Duan Y.Q., Yuan W.G., Wang X.Z. (2015). Study of Interaction between m-Nitroaniline and Bovine Serum Albumin Using Fluorospectrphotometry. PTCA.

[32] Xu H.C., Yu G.H., Yang L.Y., Jiang H.L. (2013). Combination of two-dimensional correlation spectroscopy and parallel factor analysis to characterize the binding of heavy metals with DOM in lake sediments. J. Hazard. Mater..

[33] Evans D., Dillon P. (2004). Complexation between Hg(II) and Dissolved Organic Matter in Stream Waters: An Application of Fluorescence Spectroscopy. Biogeochemistry.

[34] Hernández D., Plaza C., Senesi N., Polo A. (2006). Detection of Copper(II) and Zinc(II) binding to humic acids from pig slurry and amended soils by fluorescence spectroscopy. Environ. Pollut..

[35] Guo X., Xi B., Xie S., Ma W., Yu H., He X. (2012). Study on fluorescence spectra and UV-vis spectra of dissolved organic matter collected from sediment pore water in Wuliangsuhai Lake. Chin. J. Environ. Eng..

[36] Yang Y., Lan Y., Jin P., Xu H. (2017). Characteristic and influential factors of humic acid complexed with Cd^2+^. Environ. Chem..

[37] Tian C., Wang J., Yuan X., Liu G. (2012). Extraction and ultraviolet absorption spectra of humic acid in marine sediment. Mar. Environ. Sci..

[38] Grassi M., Rosa M. (2010). Humic acids of different origin as modifiers of cadmium-ion chemistry: A spectroscopic approach to structural properties and reactivity. Inorg. Chim. Acta.

[39] Liu J., Wang J., Chen Y., Lippold H., Lippmannpipke J. (2010). Comparative characterization of two natural humic acids in the Pearl River Basin, China and their environmental implications. J. Environ. Sci.

[40] Gondar D., Lopez R., Fiol S., Antelo J.M., Arce F. (2005). Characterization and acid–base properties of fulvic and humic acids isolated from two horizons of an ombrotrophic peat bog. Geoderma.

[41] Lan Y.Q. (2011). Study on Characteristic of Heavy Metals and Humic Acid in the Water Environment. Master’s Thesis.

